# Raising awareness of Cervical Cancer prevention: perspectives and challenges

**DOI:** 10.1016/j.lana.2022.100261

**Published:** 2022-04-21

**Authors:** The Lancet Regional Health– Americas

Cervical cancer is the fourth most common gynaecological cancer in the world. According to GLOBOCAN 2020, it has an age-standardised rate (ASR) world incidence of 13.3 per 100 000 worldwide, and 14.9 per 100 000 in Latin America and the Caribbean (LAC) region. In the LAC region, cervical cancer is the third most common cancer type among women, with the highest ASR mortality after breast and colorectum cancers. Human papillomavirus (HPV) infection is one of the main causes of cervical cancer. More than 170 HPV serotypes have been described, and HPV16 and HPV18 are the most frequently cited aetiological agents of cervical cancer. HPV infection is sexually transmitted and, despite being preventable, it still leads to cervical cancer, causing over 300 000 female deaths worldwide.

Early detection of HPV lesions, such as by Pap smear and HPV screening, is a cost-effective way to prevent cervical cancer. WHO recommends screening every 3–5 years after age 30 years in the general population of women. However, in many low-income and middle-income countries, there are barriers to accessing these tests such as fear of gynaecological exams, cultural beliefs, insurance status, or sociodemographic inequities, among others. Thus, many HPV infections remain undiagnosed. As an approach to boosting test accessibility, Shin et al., 2022 conducted a micro-costing analysis on HPV self-sampling in Peru. These tests are distributed by women recruited by the HOPE Project, who help disseminate the self-sampling kits and link women to follow-up care. The HOPE Ladies raised awareness of testing in the community and were able to increase testing, with a participation of 94.2% compared with 53.3% in the control group with practitioner-sampled tests. According to the micro-costing analysis, the programme had competitive prices and could be successfully implemented in Peru, and probably other countries in the Americas. Loss to follow-up after Pap smear should also be of concern, and a combination of self-testing and text messages to increase triage has been proven effective. For example, in Jujuy, Argentina, a study implemented text (SMS) messages to increase follow-up, resulting in a 15.5% increase in adherence among women after receiving the HPV result. SMS messages are low-cost and could be useful in increasing triage and preventing cancer.

Another highly successful approach to prevent cervical cancer is HPV vaccination, which targets mainly teenage girls and boys. Vaccination uptake varies depending on the area and time period. For example, in Canada, vaccination coverage ranges from 46.3% to 93.9% across the territories (2013-2015). In Brazil, vaccination rates decreased from 92% in 2014 to 69.5% (in girls aged 9–11 years) in 2015. In Colombia, the decline was even more pronounced: in 2013 the coverage was 97.5% and dropped to 20.4% in 2014, due to miscommunication about vaccination safety. The cost of the vaccine, misinformation, and cultural barriers are just some of the obstacles to a successful immunisation programme. In Canada, there are still issues regarding HPV vaccination rollout among children, as each province and territory is responsible for their health care, including vaccination programmes. Shapiro and colleagues evaluated the direct impact of public-funded, school-based HPV vaccination programmes for boys in Canada. Each jurisdiction implemented the vaccination programme for boys at different time points; however, some provinces did not implement the publicly funded HPV vaccination for boys, only for girls. The authors found a correlation between public-funded, school-based vaccination and greater uptake in boys, but there was no indirect uptake effect in girls. A survey was also applied to the parents to evaluate psychosocial factors and self-report vaccination, and a higher uptake was associated with child´s older age, health-care provider recommendation, and knowledge regarding vaccine effectiveness.

In 2018, WHO announced a global call for action to eliminate cervical cancer, and in 2020 the World Health Assembly adopted the Global Strategy to Accelerate the Elimination of Cervical Cancer, focusing on three main goals: one, vaccination; two, screening; and three, treatment. Latin America should build on its reputation as a region with a successful history of vaccination programme implementation, being one of the first to introduce new vaccines such as rotavirus and pneumococcal conjugate vaccines and successfully reaching coverage higher than 95% for Bacillus Calmette–Guérin (BCG) vaccine in countries such as Brazil, Argentina, Chile, and Bolivia. This historical success should be extended to HPV vaccination programmes across the region. In fact, HPV vaccine is already recommended and included as part of national immunisation programmes in many LAC countries, such as Panama and Mexico, some of the first countries that introduced HPV vaccines. Alongside the inclusion of HPV vaccines in national immunisation programmes, raising disease awareness in the population is an important task that can lead to an increase in testing and treatment seeking for HPV infection and cervical cancer. Incorporating the three goals of this Global Strategy—vaccination, screening, and treatment—into governments’ action plans will help eradicate cervical cancer sooner in LAC countries.

